# Association of Metabolic Dysfunction-Associated Fatty Liver Disease With Left Ventricular Diastolic Function and Cardiac Morphology

**DOI:** 10.3389/fendo.2022.935390

**Published:** 2022-07-19

**Authors:** Dandan Peng, Zhenqiu Yu, Mingwei Wang, Junping Shi, Lei Sun, Yuanyuan Zhang, Wenbin Zhao, Chen Chen, Jiake Tang, Chunyi Wang, Jie Ni, Wen Wen, Jingjie Jiang

**Affiliations:** ^1^ School of Clinical Medicine, Guizhou Medical University, Guiyang, China; ^2^ The Affiliated Hospital of Guizhou Medical University, Guizhou Medical University, Guiyang, China; ^3^ Hangzhou Institute of Cardiovascular Diseases, Affiliated Hospital of Hangzhou Normal University, Hangzhou Normal University, Hangzhou, China; ^4^ Department of Hepatology, The Affiliated Hospital and Institute of Hepatology and Metabolic Disease, Hangzhou Normal University, Hangzhou, China; ^5^ Department of Cardiovascular Ultrasonic Center, First Affiliated Hospital, College of Medicine, Zhejiang University, Hangzhou, China

**Keywords:** metabolic dysfunction-associated fatty liver disease, cardiac remodeling, left ventricular diastolic dysfunction, type 2 diabetes mellitus, obesity

## Abstract

**Background and Aim:**

Non-alcoholic fatty liver disease (NAFLD) is closely related to cardiovascular diseases (CVD). A newly proposed definition is metabolic dysfunction-associated fatty liver disease (MAFLD), which was changed from NAFLD. The clinical effect of this change on abnormalities of cardiac structure and function is yet unknown. We aimed to examine whether MAFLD is associated with left ventricular (LV) diastolic dysfunction (LVDD) and cardiac remolding and further identify the impact of different subgroups and severity of MAFLD.

**Method:**

We evaluated 228 participants without known CVDs. Participants were categorized by the presence of MAFLD and the normal group. Then, patients with MAFLD were subclassified into three subgroups: MAFLD patients with diabetes (diabetes subgroup), overweight/obesity patients (overweight/obesity subgroup), and lean/normal-weight patients who had two metabolic risk abnormalities (lean metabolic dysfunction subgroup). Furthermore, the severity of hepatic steatosis was assessed by transient elastography (FibroScan^®^) with a controlled attenuation parameter (CAP), and patients with MAFLD were divided into normal, mild, moderate, and severe hepatic steatosis groups based on CAP value. Cardiac structure and function were examined by echocardiography.

**Results:**

LVDD was significantly more prevalent in the MAFLD group (24.6% vs. 60.8%, *p* < 0.001) compared to the normal group. The overweight subgroup and diabetes subgroup were significantly associated with signs of cardiac remolding, including interventricular septum thickness, LV posterior wall thickness, left atrial diameter (all *p* < 0.05), relative wall thickness, and LV mass index (all *p* < 0.05). Additionally, moderate-to-to severe steatosis patients had higher risks for LVDD and cardiac remolding (all *p*-values < 0.05).

**Conclusion:**

MAFLD was associated with LVDD and cardiac remolding, especially in patients with diabetes, overweight patients, and moderate-to-to severe steatosis patients. This study provides theoretical support for the precise prevention of cardiovascular dysfunction in patients with MAFLD.

## Introduction

Non-alcoholic fatty liver disease (NAFLD) has become a common metabolic disease worldwide, with the estimated prevalence of a quarter of the population (1) and is an independent risk factor for cardiovascular diseases (CVDs) (2, 3). It is a clinicopathological syndrome characterized by diffuse hepatocellular bullae fat, excluding excessive alcohol consumption and other clearly defined causes of liver damage (4). In 2020, an international panel of experts reached a consensus to change the name from NAFLD to metabolic dysfunction-associated fatty liver disease (MAFLD); this new definition does not require exclusion of patients with alcohol consumption, or other chronic liver diseases, and the presence of metabolic abnormalities in lean and normal-weight fatty liver patients and is a more appropriate overarching term than the former name NAFLD (5, 6). Therefore, the renaming of fatty liver diseases may have different effects on the results of some clinical studies (7).

Importantly, the main cause of death in patients with NAFLD is CVDs, rather than hepatic causes (8). MAFLD is a kind of heterogeneous disease, which can be categorized into different subtypes based on the inclusion criteria, and the effects on CVDs might be different in MAFLD subtypes (9). Abnormalities of left ventricular (LV) diastolic function and cardiac structure may have no obvious clinical manifestations in the early stage, but the progression of the disease can induce heart failure or other life-threatening cases. In recent years, some studies have investigated whether hepatic adipose deposition has adverse effects on cardiac structure and function, especially research on a relationship between different subtypes and severity of fatty liver and risk of CVDs (10–12). There are differences in long-term outcomes among MAFLD patients with different diagnostic criteria, and some subtypes may have higher risks of all-cause mortality, which is of great significance for the precise prevention of poor prognosis (9). However, the correlation between MAFLD and abnormalities of LV diastolic function and cardiac structure is lacking. In this study, Doppler echocardiography was used to assess differences in LV structure and diastolic function among subtypes and the severity of MAFLD patients. It is helpful to identify early intervention and actively monitor high-risk population groups to avoid serious heart damage.

## Methods

### Study Participants

This cross-sectional study population consisted of patients who visit the metabolic disease center in Hangzhou Normal University Affiliated Hospital and were enrolled between March 2021 and May 2022 in the Hangzhou Normal University Affiliated Hospital for health examinations. The baseline characteristics were compared among the groups. All subjects were assessed by imaging techniques to investigate the clinical association between MAFLD and LV diastolic dysfunction (LVDD) and cardiac remolding. This study protocol and analysis of the data were approved by the institutional review board of the hospital.

**Figure 1:**
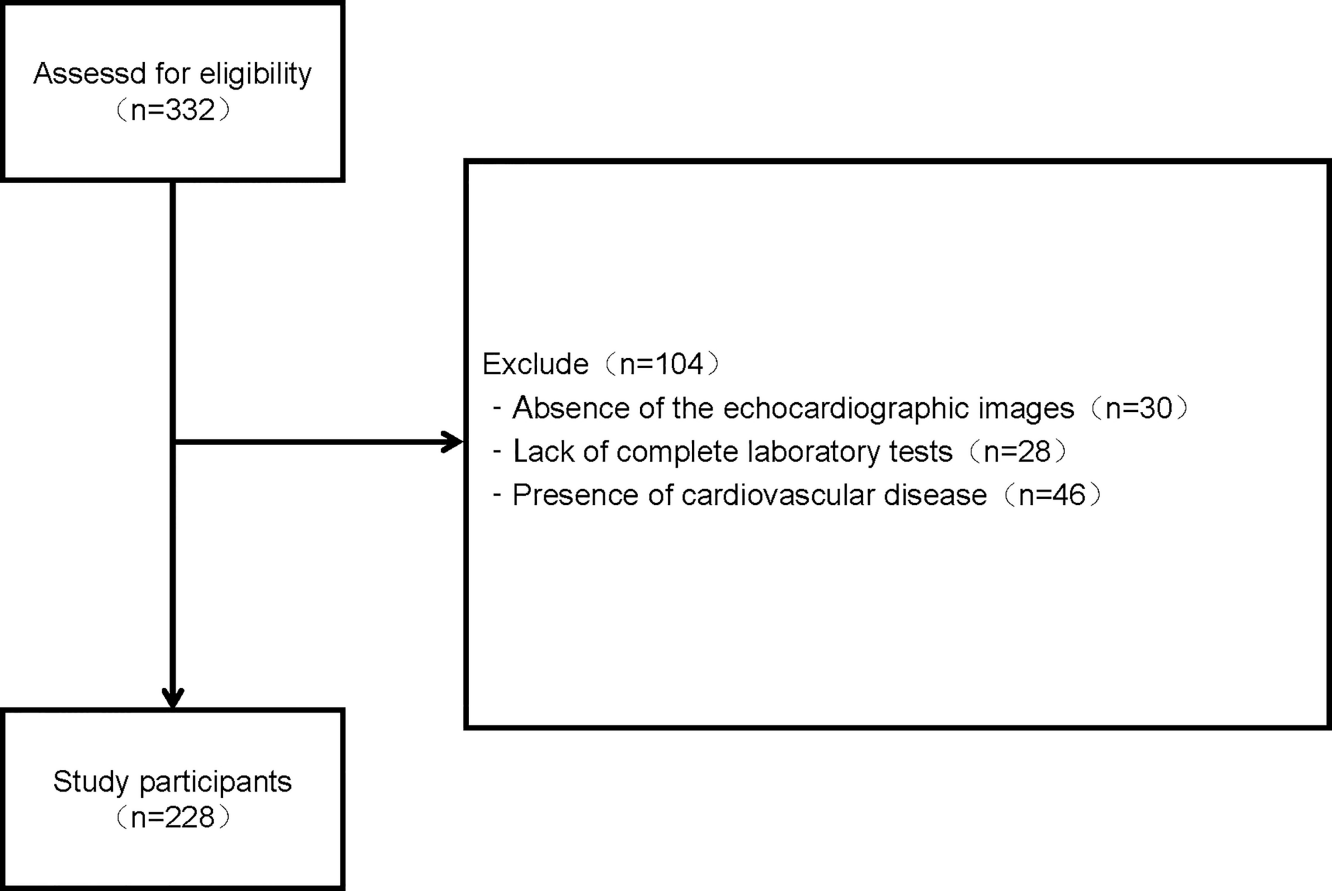
Flowchart of enrolled study participants.

A total of 332 participants were initially evaluated, 30 (9.04%) individuals were excluded because of the absence of the echocardiographic images, 28 (8.43%) individuals were removed because of lack of complete laboratory tests, and 46 (13.86%) individuals were excluded because of history of CVDs including heart failure, ischemic heart disease, severe arrhythmia, and moderate or severe valvular heart disease. Finally, a total number of 228 subjects were included in this study. The evaluation of screening programs is presented in **Figure 1**.

### Clinical Assessment and Laboratory Measurements

Demographics, medical history, waist circumference, and social habits including smoking and alcohol consumption, were obtained *via* an outpatient collection at the first visit. Smoking status was categorized into never, past, or current smoking. Current smoking was defined as having smoked at least 1 cigarette per day; history of alcohol consumption was categorized into never, past, little drinking (1–19 g/day), moderate drinking (20–39 g/day), and excessive drinking (≥40 g/day). Body mass index (BMI) was calculated as weight (kg) divided by the square of the height (m). Waist circumference was measured at the midpoint between the lower ribs and the iliac crest after normal expiration. Blood pressure was measured on both sides and read by a mercury sphygmomanometer after at least 5 min of rest, and systolic and diastolic blood pressure was recorded. Blood samples were collected from all participants after overnight fasting to determine laboratory parameters, such as cholesterol level, plasma glucose, related indexes of liver function, and inflammation.

Hypertension was defined as blood pressure ≥ 140/90 mmHg and/or current antihypertensive therapy. Diabetes was defined according to the 2020 China Guideline for Type 2 Diabetes. It mainly includes typical diabetes symptoms and one of the following conditions: fasting plasma glucose (FPG) level ≥7.0 mmol/L or random and postprandial blood glucose level ≥11.1 mmol/L or glycosylated hemoglobin (HbA1c) level ≥6.5%. Participants with a previous history of diabetes or the current use of glucose-lowering agents were also regarded as current diabetic patients.

### Measurements of Hepatic Steatosis and Fibrosis

Abdominal ultrasound (Philips Epiq 7C Color Doppler ultrasound diagnostic instrument) or transient elastography (FibroScan^®^ 501, Echosens, Paris, France) was used to diagnose fatty liver, and it was also confirmed that it was done by trained radiologists who were blinded to the data of all participants, including general information, laboratory data, and echocardiography. The severity of hepatic steatosis was estimated using controlled attenuation parameter (CAP) values, which were examined by FibroScan^®^. According to CAP value, the severity of fatty liver was categorized into three grades, which have respectively established cutoff values of 248, 268, and 280 dB/m for >S0, >S1, and >S2 and described as mild, moderate, or severe hepatic steatosis (13). The liver stiffness measurements (LSMs) in each patient were also measured, and their median value was computed. The LSM was stated in kilopascals. For the LSM cut-off value, ≥8.0 kPa is used for ruling in liver fibrosis (14).

### Diagnosis of Metabolic Dysfunction-Associated Fatty Liver Disease

MAFLD was diagnosed by the international expert consensus statement in 2020 (15). The criteria include evidence of hepatic steatosis and meanwhile complicated with any one of the following three conditions: overweight/obesity (BMI ≥ 23 kg/m^2^), presence of type 2 diabetes mellitus (T2DM), or lean/normal subjects with metabolic dysregulation. Metabolic dysfunction was defined as the concurrence of at least two metabolic risk abnormalities (**Table 1**).

**Table 1:** The inclusion criteria of metabolic risk abnormalities.

The inclusion criteria of metabolic risk abnormalities
− Waist circumference ≥90/80 cm in men and women − SBP ≥ 130 mmHg and DBP ≥ 85 mmHg; antihypertensive therapy − Plasma triglycerides ≥150 mg/dl (≥1.70 nmol/L); lipid-lowering drug therapy − Plasma HDL-cholesterol <40 mg/dl (<1.0 mmol/L) and <50 mg/dl (<1.3 mmol/L) respectively for men and women; specific drug treatment − Diagnosis of prediabetes or HbA1c 5.7% to 6.4% − Homeostasis model assessment (HOMA)—insulin resistance score ≥ 2.5 − Plasma high-sensitivity C-reactive protein (h-CRP) level > 2 mg/L

SBP, systolic blood pressure; DBP, diastolic blood pressure; HDL-cholesterol, high-density lipoprotein cholesterol; HbA1c, hemoglobin A1c.

### Subgroups of Metabolic Dysfunction-Associated Fatty Liver Disease

In this study, subgroups of MAFLD were classified by two methods. First, patients with MAFLD were divided into three subgroups according to their inclusion criteria, including diabetic patients (diabetes subgroup), non-diabetes but overweight/obesity patients (overweight/obesity subgroup), and lean/normal-weight patients who had two metabolic risk abnormalities (lean metabolic dysfunction subgroup). Since the histological steatosis severity is closely associated with CVD (16, 17), patients with MAFLD were divided into three grades—mild, moderate, and severe—based on the CAP cutoff value, as previously described.

### Echocardiography

All participants were managed by professional sonographers using a Philips Epiq 7C Color Doppler Ultrasound diagnostic instrument, with X5-1 (1~5 MHz) probe. Before the examination, the patients were instructed to be quiet for at least 15 min while in a supine position or left decubitus position and to perform calm breathing. The measurement method was in accordance with the Chinese Adult Echocardiography Measurement guidelines. The images were analyzed by another experienced echocardiographer, blinded to who has hepatic steatosis. The parameters of cardiac structure, including left atrial diameter (LAD), LV end-diastolic diameter (LVEDD), interventricular septum thickness (IVST), and LV posterior wall thickness (LVPWT) were routinely measured. LV mass (LVM) was calculated with following formula: LVM= 0.8 X {1.04[(LVEDD+IVST+PWT)3 -(LVEDD)3 ]} + 0.6 g(18). Body surface area (BSA) was calculated by the following formula: 0.0061 × Height + 0.0124 × Weight − 0.0099. LVM index (LVMI) was calculated as LVM divided by the BSA. LV end-diastolic volume (LVEDV) is calculated by the following formula: 7.0/(2.4 + LVEDD) × LVEDD^3^. The relative wall thickness (RWT) was calculated by the formula (2 × PWT)/LVEDD, and increased RWT was defined as RWT > 0.42 (19). Peak velocities of the early (E) and late (A) phases of the mitral inflow were also measured, and an E/A ratio < 1 was considered as decreased diastolic function and defined as cardiac insufficiency. The LVDD was diagnosed by sonographers, and its prevalence was recorded.

### Statistical Analysis

**Figure 2:**
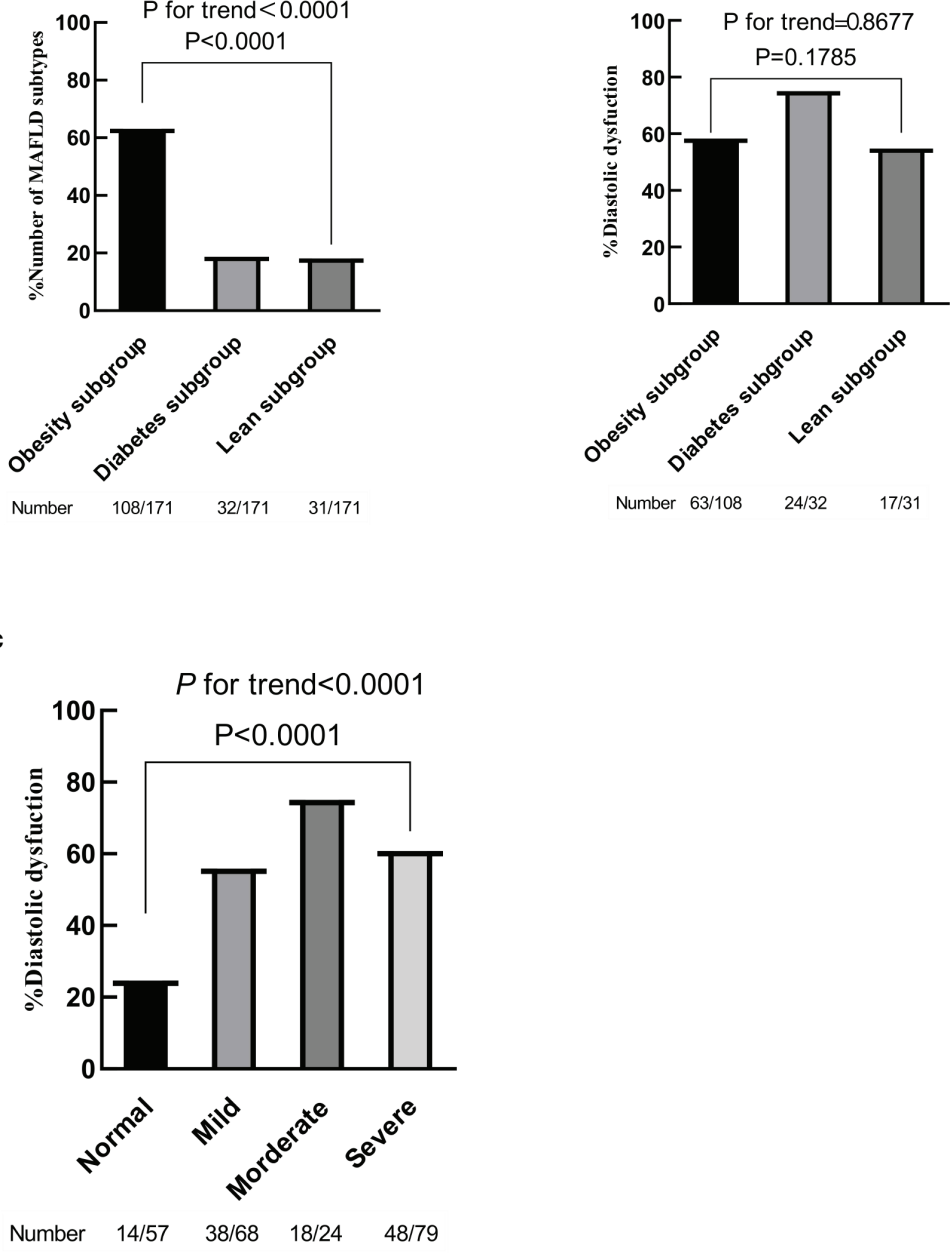
Prevalence of different metabolic dysfunction-associated fatty liver disease (MAFLD) subgroups **(A)**. Prevalence of left ventricular diastolic dysfunction according to MAFLD subtypes **(B)**. Prevalence of left ventricular diastolic dysfunction according to the degree of hepatic steatosis **(C)**. *p* for trend by chi-square test for linear-by-linear association.

All continuous variables were expressed as mean and SD (mean ± SD). The proportions for categorical variables were represented as the number of cases in each category and percentages. Two data groups were compared using Student’s t-test and the ANOVA test when it was necessary to compare at least three data groups for continuous variables. The prevalence of LVDD was presented as the number of cases in each category and percentage using the chi-square test for linear-by-linear association. Additionally, the trend in the proportion of each subgroup in MAFLD and the prevalence of LVDD in MAFLD subgroups were analyzed using the Jonckheere–Terpstra test (**Figure 2**).

The analysis of the impact of hepatic steatosis on the cardiac structure was done in 2 steps. First, differences in the echocardiographic parameters between groups of patients with hepatic steatosis and no steatosis were analyzed using Student’s t-test (**Table 2**) and comparison between subgroups of MAFLD using the ANOVA test (**Tables 3**, **4**). Meanwhile, the relationship between liver fibrosis and cardiac structure was analyzed in **Table 5**. Second, a series of multivariable linear regression analyses were applied to assess the influence of different subgroups of MAFLD on echocardiographic parameters of cardiac structure with a 95% CI, after controlling for potential confounding factors. Model 2 was adjusted for age, sex, smoking, alcohol consumption, BMI and hypertension and further adjusted for alanine transaminase (ALT), aspartate transaminase (AST), and total cholesterol (TC) in Model 3. The *p*-values in all cases were calculated, and it was considered that there is a statistically significant difference between the means of the compared groups when the *p*-value was less than 0.05. Statistical analyses were performed using SPSS version 21.0 software (IBM Corp., Armonk, NY, USA).

**Table 2:** Characteristics of the study samples.

Characteristics	Normal	MAFLD	*p*-Value
	(N = 57)	(N = 171)	
**Age (years)**	40.89 ± 12.91	48.84 ± 11.64	**<0.001**
**Sex (female/male)** Male Female	33 (57.9%) 24 (42.1%)	121 (70.8%) 50 (29.2%)	0.102
**Type 2 diabetes mellitus (%)**	2 (3.5%)	32 (18.7%)	**0.004**
**Hypertension (%)**	18 (31.6%)	105 (61.4%)	**<0.001**
**Smoking habit** Never Presence Quitting	2 (3.5%)	32 (18.7%)	**0.004**
**Dairy alcohol consumption (g/day)** 0 1–19 20–39 ≥40	45 (78.9%) 10 (17.5%) 0 (0%) 2 (3.5%)	125 (73.1%) 39 (22.8%) 3 (1.8%) 4 (2.3%)	0.579
**Height (cm)**	167.01 ± 9.21	167.19 ± 7.17	0.894
**Weight (kg)**	65.07 ± 11.87	71.73 ± 11.28	**<0.001**
**Body mass index (kg/m^2^)**	23.12 ± 3	25.57 ± 3.05	**<0.001**
**Waist circumference (cm)**	83.56 ± 11.52	92 ± 9.01	**<0.001**
**Left SBP (mmHg)**	125.36 ± 18.61	137.56 ± 20.48	**<0.001**
**Left DBP (mmHg)**	83.47 ± 10.99	90.94 ± 13.08	**<0.001**
**Right SBP (mmHg)**	127.24 ± 18.83	139.45 ± 20.78	**<0.001**
**Right DBP (mmHg)**	85.12 ± 11.11	92.06 ± 12.9	**<0.001**
**Red blood cell count (×10^12^/L)**	4.76 ± 0.58	4.89 ± 0.45	0.118
**MCHC (g/L)**	331.75 ± 29.8	336.89 ± 10.6	0.207
**White blood cell count (×10^9^/L)**	5.85 ± 1.3	6.45 ± 1.71	**0.017**
**Neutrophil count (×10^9^/L)**	3.34 ± 1.08	3.95 ± 1.43	**0.001**
**Platelet count (×10^9^/L)**	230.78 ± 47.2	229.46 ± 70.61	0.895
**ALT (U/L)**	25.43 ± 23.16	33.2 ± 28.26	0.062
**AST (U/L)**	24.21 ± 10.03	27.4 ± 24.21	0.334
**Lactate dehydrogenase (U/L)**	169.42 ± 34.05	186.52 ± 76.03	0.102
**γ-GGT (U/L)**	29.4 ± 30.05	39.53 ± 39.39	0.077
**ALP (U/L)**	75.8 ± 26.61	84.28 ± 20.16	**0.012**
**Total protein (g/L)**	72.31 ± 3.46	71.11 ± 4.58	**0.041**
**Albumin (g/L)**	44.59 ± 3.36	43.66 ± 3.25	0.065
**Globulin (g/L)**	27.54 ± 4.3	27.33 ± 3.24	0.701
**Total bilirubin (μmol/L)**	16.54 ± 8.41	15.07 ± 5.62	0.222
**Triglyceride (mmol/L)**	1.18 ± 0.73	1.91 ± 1.18	**<0.001**
**Total cholesterol (mmol/L)**	4.89 ± 1.07	4.57 ± 1.08	0.055
**HDL‐cholesterol (mmol/L)**	1.35 ± 0.26	1.13 ± 0.31	**<0.001**
**LDL‐cholesterol (mmol/L)**	2.89 ± 0.72	2.76 ± 0.75	0.262
**Fasting blood glucose (mmol/L)**	5.42 ± 0.51	6.27 ± 1.87	**<0.001**
**BUN (mmol/L)**	4.93 ± 1.13	5.27 ± 1.46	0.116
**Creatinine (mmol/L)**	68.6 ± 16.01	73.86 ± 18.58	0.057
**Uric acid (μmol/L)**	330 ± 75.18	373.59 ± 89.06	**<0.001**
**CRP (mg/L)**	1.08 ± 1.48	2 ± 4.29	**0.017**
**HbA1c (%)**	5.55 ± 0.4	5.96 ± 1.04	**<0.001**
**CAP (dB/m)**	219.75 ± 20.31	287.4 ± 34.79	**<0.001**
**LSM (kPa)**	4.55 ± 0.92	5.27 ± 1.33	**<0.001**
**IVST (mm)**	0.93 ± 0.11	1.05 ± 0.14	**<0.001**
**LVPWT (mm)**	0.89 ± 0.1	0.98 ± 0.1	**<0.001**
**LVEDD (mm)**	4.57 ± 0.4	4.73 ± 0.38	**0.010**
**LA diameter (mm)**	3.21 ± 0.41	3.39 ± 0.41	**0.004**
**RWT**	0.39 ± 0.04	0.41 ± 0.05	**<0.001**
**LVEDV (ml)**	97.3 ± 19.81	104.98 ± 19.83	**0.012**
**LVM (g)**	142.02 ± 34.75	172.64 ± 37.22	**<0.001**
**LVMI (BSA)**	78.06 ± 17.28	90.91 ± 18.18	**<0.001**
**LVDD (%)**	14 (24.6%)	104 (60.8%)	**<0.001**

Values are mean ( ± SD). Statistically significant values are highlighted in bold (P<0.05).

SBP, systolic blood pressure; DBP, diastolic blood pressure; MCHC, mean corpuscular hemoglobin concentration; AST, aspartate aminotransferase; ALT, alanine aminotransferase; γ-GGT, gamma-glutamyl transpeptidase; ALP, alkaline phosphatase; HDL-cholesterol, high-density lipoprotein cholesterol; LDL-cholesterol, low-density lipoprotein cholesterol; CRP, C-reactive protein; HbA1c, hemoglobin A1c; CAP, controlled attenuation parameter; LSM, liver stiffness measurements; BSA, body surface area; IVST, interventricular septum thickness; LVPWT, left ventricular posterior wall thickness; LA, left atrial; RWT, relative wall thickness; LVEDD, left ventricular end-diastolic diameter; LVM, left ventricular mass; LVMI, left ventricular mass index; LVDD, left ventricular diastolic dysfunction.

**Table 3:** Characteristics of different MAFLD subgroups.

	MAFLD		
Characteristics	Obesity subgroup	Diabetes subgroup	Lean subgroup	*p*-Value
	(N = 108)	(N = 32)	(N = 31)	
**Age (years)**	46.47 ± 10.87	55.84 ± 10.37	49.9 ± 12.72	<0.001^*+^
**Sex (female/male)** Male Female	79 (73.1%) 29 (26.9%)	25 (78.1%) 7 (21.9%)	17 (54.8%) 14 (45.2%)	0.085
**Type 2 diabetes mellitus (%)**	0 (0%)	32 (100%)	0 (0%)	<0.001^*#+^
**Hypertension (%)**	70 (64.8%)	20 (62.5%)	15 (48.4%)	0.251
**Smoking habit** Never Presence Quitting	76 (70.4%) 26 (24.1%) 6 (5.4%)	25 (78.1%) 7 (21.9%) 0 (0%)	25 (80.6%) 6 (19.4%) 0 (0%)	0.380
**Dairy alcohol consumption (g/day)** 0 1–19 20–39 ≥40	75 (69.4%) 27 (25.0%) 2 (1.9%) 4 (3.7%)	22 (68.8%) 9 (28.1%) 1 (3.1%) 0 (0%)	28 (90.3%) 3 (9.7%) 0 (0%) 0 (0%)	0.258
**Height (cm)**	167.52 ± 6.59	166.96 ± 7.09	166.25 ± 9.13	0.675
**Weight (kg)**	74.92 ± 9.6	72.41 ± 12.15	59.91 ± 7.63	**<0.001^#+^**
**Body mass index (kg/m^2^)**	26.63 ± 2.41	25.85 ± 3.07	21.61 ± 1.41	**<0.001^#+^**
**Waist circumference (cm)**	94.72 ± 7.76	91.31 ± 9.4	83.22 ± 6.82	<0.001^*#+^
**Left SBP (mmHg)**	138.02 ± 20.53	141.31 ± 21.09	132.09 ± 19.18	0.189
**Left DBP (mmHg)**	92.3 ± 13.02	90.56 ± 14.49	86.61 ± 11.05	**0.010^#^**
**Right SBP (mmHg)**	139.55 ± 21.09	142.96 ± 20.96	135.48 ± 19.42	0.361
**Right DBP (mmHg)**	93.42 ± 13.45	92.28 ± 12.27	87.09 ± 10.47	0.054
**Red blood cell count (×10^12^/L)**	4.97 ± 0.42	4.86 ± 0.5	4.66 ± 0.42	**0.003^#^**
**MCHC (g/L)**	337.24 ± 10.53	337.21 ± 12.81	335.35 ± 8.26	0.673
**White blood cell count (×10^9^/L)**	6.55 ± 1.78	6.51 ± 1.54	6.03 ± 1.63	0.324
**Neutrophil count (×10^9^/L)**	3.99 ± 1.5	3.96 ± 1.12	3.84 ± 1.5	0.880
**Platelet count (×10^9^/L)**	233.61 ± 80.02	217.59 ± 48.18	227.25 ± 52.9	0.523
**ALT (U/L)**	35.74 ± 27.35	36.4 ± 39.12	21.06 ± 9.52	**0.029^#+^**
**AST (U/L)**	29.01 ± 28.59	27.12 ± 17.99	22.09 ± 5.71	0.375
**Lactate dehydrogenase (U/L)**	191.75 ± 91.08	175.06 ± 30.01	180.12 ± 44.27	0.485
**γ-GGT (U/L)**	42.09 ± 33.9	44.43 ± 63.71	25.54 ± 15.81	0.088
**ALP (U/L)**	83.34 ± 19.38	92.03 ± 22.85	79.54 ± 18.23	0.035^*+^
**Total protein (g/L)**	71.58 ± 4.51	69.64 ± 4.84	71 ± 4.31	0.106
**Albumin (g/L)**	44.01 ± 2.79	42.96 ± 3.15	43.16 ± 4.54	0.176
**Globulin (g/L)**	27.57 ± 3.19	26.66 ± 3.69	27.19 ± 2.87	0.366
**Total bilirubin (μmol/L)**	15.3 ± 5.95	14.31 ± 4.18	15.04 ± 5.85	0.686
**Triglyceride (mmol/L)**	2.01 ± 1.22	1.75 ± 0.91	1.7 ± 1.28	0.302
**Total cholesterol (mmol/L)**	4.64 ± 1.07	4.26 ± 1.14	4.65 ± 1.04	0.204
**HDL‐cholesterol (mmol/L)**	1.1 ± 0.23	1.05 ± 0.2	1.3 ± 0.55	**0.003^#+^**
**LDL‐cholesterol (mmol/L)**	2.84 ± 0.7	2.54 ± 0.83	2.7 ± 0.82	0.121
**Fasting glucose (mmol/L)**	5.72 ± 0.76	8.62 ± 3.02	5.76 ± 1.08	<0.001^*+^
**BUN (mmol/L)**	5.14 ± 1.51	5.74 ± 1.51	5.22 ± 1.15	0.122
**Creatinine (mmol/L)**	75.52 ± 19.07	71.99 ± 17.67	70 ± 17.54	0.284
**Uric acid (μmol/L)**	395.15 ± 92.8	343.28 ± 71.17	329.8 ± 66.04	<0.001^*#^
**CRP (mg/L)**	1.81 ± 2.83	2.64 ± 6.65	2.03 ± 5.41	0.633
**HbA1c (%)**	5.6 ± 0.39	7.55 ± 1.47	5.61 ± 0.34	<0.001^*+^
**CAP (dB/m)**	292.29 ± 34.3	286.84 ± 36.4	270.96 ± 30.49	**0.010^#^**
**LSM (kPa)**	5.34 ± 1.47	5.55 ± 1.06	4.72 ± 0.87	**0.029^#+^**
**Visceral fat (cm^2^)**	82.67 ± 34.01	88.59 ± 24	56.7 ± 17.58	**<0.001^#+^**
**Abdominal subcutaneous fat (cm^2^)**	199.26 ± 73.47	161.9 ± 64.72	130.48 ± 36.11	<0.001^*#^
**IVST (mm)**	1.04 ± 0.12	1.13 ± 0.2	0.97 ± 0.11	<0.001^*#+^
**LVPWT (mm)**	0.99 ± 0.09	1.04 ± 0.09	0.89 ± 0.1	<0.001^*#+^
**LVEDD (mm)**	4.76 ± 0.37	4.77 ± 0.32	4.56 ± 0.4	**0.024^#+^**
**LA diameter (mm)**	3.45 ± 0.41	3.45 ± 0.37	3.14 ± 0.37	**0.001^#+^**
**RWT**	0.41 ± 0.04	0.44 ± 0.05	0.39 ± 0.04	0.001^*#+^
**LVEDV (ml)**	106.87 ± 19.9	106.68 ± 16.96	96.63 ± 20.75	**0.034^#+^**
**LVM (g)**	174.89 ± 34.02	190.9 ± 34.06	145.96 ± 37.61	<0.001^*#+^
**LVMI (BSA)**	90.26 ± 17.19	100.46 ± 17.01	83.32 ± 19	0.001^*+^
**LVDD (%)**	63 (58.3%)	24 (75.0%)	17 (54.8%)	0.179

Values are mean ( ± SD). Statistically significant values are highlighted in bold (P<0.05).

SBP, systolic blood pressure; DBP, diastolic blood pressure; MCHC, mean corpuscular hemoglobin concentration; AST, aspartate aminotransferase; ALT, alanine aminotransferase; γ-GGT, gamma-glutamyl transpeptidase; ALP, alkaline phosphatase; HDL-cholesterol, high-density lipoprotein cholesterol; LDL-cholesterol, low-density lipoprotein cholesterol; CRP, C-reactive protein; HbA1c, hemoglobin A1c; CAP, controlled attenuation parameter; LSM, liver stiffness measurements; BSA, body surface area; IVST, interventricular septum thickness; LVPWT, left ventricular posterior wall thickness; LA, left atrial; RWT, relative wall thickness; LVEDD, left ventricular end-diastolic diameter; LVM, left ventricular mass; LVMI, left ventricular mass index; LVDD, left ventricular diastolic dysfunction.

^*^p < 0.05 obesity subgroup vs. diabetes subgroup.

^#^p < 0.05 obesity subgroup vs. lean subgroup.

^+^p < 0.05 diabetes subgroup vs. lean subgroup.

**Table 4:** Characteristics of different degrees of MAFLD patients (CAP subgroup).

Characteristics	Normal	Mild	Moderate	Severe	*p*-Value
	CAP < 248	248 ≤ CAP < 268	268 ≤ CAP ≤ 80	CAP > 280	
	(N = 57)	(N = 68)	(N = 24)	(N = 79)	
**Age (years)**	40.89 ± 12.91	49.22 ± 12.03	51.58 ± 10.50	47.69 ± 11.6	**<0.001**
**Sex (female/male)** Male Female	33 (57.9%) 24 (42.1%)	47 (69.1%) 21 (30.9%)	14 (58.3%) 10 (41.7%)	60 (75.9%) 19 (24.1%)	0.113
**Type 2 diabetes mellitus (%)**	2 (3.5%)	12 (17.6%)	5 (20.8%)	15 (19.0%)	**0.047**
**Hypertension (%)**	18 (31.6%)	39 (57.4%)	15 (62.5%)	51 (64.6%)	**0.001**
**Smoking habit** Never Presence Quitting	49 (86.0%) 7 (12.3%) 1 (1.8%)	52 (76.5%) 15 (22.1%) 1 (1.5%)	19 (79.2%) 4 (16.7%) 1 (4.2%)	55 (69.6%) 20 (25.3%) 4 (5.1%)	0.397
**Dairy alcohol consumption (g/day)** 0 1–19 20–39 ≥40	45 (78.9%) 10 (17.5%) 0 (0%) 2 (3.5%)	53 (77.9%) 14 (20.6%) 0 (0%) 1 (1.5%)	19 (79.2%) 4 (16.7%) 1 (4.2%) 0 (0%)	53 (67.1%) 21 (26.6%) 2 (2.5%) 3 (3.8%)	0.521
**Height (cm)**	167.01 ± 9.21	167.85 ± 7.77	162.87 ± 6.00	167.93 ± 6.56	**0.044**
**Weight (kg)**	65.07 ± 11.87	68.46 ± 9.68	65.45 ± 8.56	76.45 ± 11.46	**<0.001**
**Body mass index (kg/m^2^)**	23.12 ± 3	24.24 ± 2.5	24.67 ± 2.86	27 ± 2.94	**<0.001**
**Waist circumference (cm)**	83.56 ± 11.52	88.14 ± 7.85	88.16 ± 7.2	96.48 ± 8.41	**<0.001**
**Left SBP (mmHg)**	125.36 ± 18.61	137.08 ± 19.09	141.08 ± 24.77	136.91 ± 20.4	**0.002**
**Left DBP (mmHg)**	83.47 ± 10.99	90.32 ± 11.57	89.33 ± 13.22	91.97 ± 14.3	**0.001**
**Right SBP (mmHg)**	127.24 ± 18.83	139.36 ± 18.55	141.87 ± 25.96	138.79 ± 21.09	**0.002**
**Right DBP (mmHg)**	85.12 ± 11.11	91.82 ± 11.27	90.04 ± 13.92	92.88 ± 13.95	**0.001**
**Red blood cell count (×10^12^/L)**	4.76 ± 0.58	4.78 ± 0.47	4.76 ± 0.46	5.03 ± 0.4	**0.002**
**MCHC (g/L)**	331.75 ± 29.8	336.08 ± 10.54	335.58 ± 11.85	337.98 ± 10.29	0.233
**White blood cell count (×10^9^/L)**	5.85 ± 1.3	6.16 ± 1.39	5.94 ± 1.51	6.85 ± 1.93	**0.001**
**Neutrophil count (×10^9^/L)**	3.34 ± 1.08	3.75 ± 1.12	3.89 ± 1.65	4.15 ± 1.58	**0.007**
**Platelet count (×10^9^/L)**	230.78 ± 47.2	229.27 ± 92.01	221.45 ± 52.85	232.05 ± 52.38	0.870
**ALT (U/L)**	25.43 ± 23.16	30 ± 29.34	24.87 ± 13.04	38.49 ± 29.87	**0.021**
**AST (U/L)**	24.21 ± 10.03	29.33 ± 36.07	23.04 ± 6.63	27.07 ± 11.73	0.454
**Lactate dehydrogenase (U/L)**	169.42 ± 34.05	191.51 ± 114.56	185.87 ± 29.48	182.43 ± 32.13	0.347
**γ-GGT (U/L)**	29.4 ± 30.05	31.66 ± 21.46	32.87 ± 24.65	48.32 ± 51.59	**0.015**
**ALP (U/L)**	75.8 ± 26.61	84.54 ± 20.25	88.08 ± 17.43	82.89 ± 20.91	0.088
**Total protein (g/L)**	72.31 ± 3.46	70.74 ± 4.94	70.74 ± 4.32	71.55 ± 4.33	0.223
**Albumin (g/L)**	44.59 ± 3.36	43.15 ± 4.02	43.45 ± 2.87	44.16 ± 2.51	0.089
**Globulin (g/L)**	27.54 ± 4.3	27.29 ± 3.23	27.28 ± 2.89	27.37 ± 3.38	0.977
**Total bilirubin (μmol/L)**	16.54 ± 8.41	14.71 ± 5.06	15.52 ± 7.55	15.24 ± 5.47	0.322
**Triglyceride (mmol/L)**	1.18 ± 0.73	1.92 ± 1.28	1.6 ± 1.08	1.99 ± 1.12	**<0.001**
**Total cholesterol (mmol/L)**	4.89 ± 1.07	4.46 ± 0.98	4.5 ± 1.3	4.69 ± 1.1	0.141
**HDL‐cholesterol (mmol/L)**	1.35 ± 0.26	1.19 ± 0.41	1.14 ± 0.3	1.07 ± 0.19	**<0.001**
**LDL‐cholesterol (mmol/L)**	2.89 ± 0.72	2.61 ± 0.69	2.73 ± 0.95	2.9 ± 0.72	0.058
***Fasting blood glucose* (mmol/L)**	5.42 ± 0.51	6.46 ± 2.37	6.16 ± 1.5	6.14 ± 1.45	**0.004**
**BUN (mmol/L)**	4.93 ± 1.13	5.25 ± 1.28	5.24 ± 1.43	5.29 ± 1.63	0.531
**Creatinine (mmol/L)**	68.6 ± 16.01	73.6 ± 19.54	71.22 ± 17.57	74.88 ± 18.18	0.338
**Uric acid (μmol/L)**	330 ± 75.18	351.08 ± 81.09	378.41 ± 84.65	391.51 ± 93.55	**0.001**
**CRP (mg/L)**	1.08 ± 1.48	1.29 ± 2.4	3.72 ± 7.99	2.1 ± 3.86	**0.023**
**HbA1c (%)**	5.55 ± 0.4	5.98 ± 1.03	6.03 ± 0.94	5.93 ± 1.09	**0.036**
**CAP (dB/m)**	219.75 ± 20.31	255.83 ± 6.01	272.75 ± 2.62	319.03 ± 25.64	**<0.001**
**LSM (kPa)**	4.55 ± 0.92	5 ± 1.01	5.27 ± 1.22	5.5 ± 1.56	**0.001**
**IVST (mm)**	0.93 ± 0.11	1.02 ± 0.13	1.05 ± 0.15	1.07 ± 0.15	**<0.001**
**LVPWT (mm)**	0.89 ± 0.1	0.96 ± 0.11	0.96 ± 0.1	1.01 ± 0.1	**<0.001**
**LVEDD (mm)**	4.57 ± 0.4	4.72 ± 0.39	4.68 ± 0.47	4.75 ± 0.33	0.126
**LA diameter (mm)**	3.21 ± 0.41	3.29 ± 0.35	3.48 ± 0.70	**3.46 ± 0.32**	0.002
**RWT**	0.39 ± 0.04	0.4 ± 0.04	0.41 ± 0.06	0.42 ± 0.04	**0.026**
**LVEDV (ml)**	97.3 ± 19.81	104.74 ± 20.74	103.28 ± 25.75	105.7 ± 17.04	0.16
**LVM (g)**	142.02 ± 34.75	167.21 ± 39.81	167.68 ± 36.49	178.83 ± 34.55	**<0.001**
**LVMI (BSA)**	78.06 ± 17.28	89.74 ± 20.31	93.55 ± 19.85	91.11 ± 15.7	**0.004**
**LVDD (%)**	14 (24.6%)	38 (55.9%)	18 (75.0%)	48 (60.8%)	**<0.001**

Values are mean ( ± SD). Statistically significant values are highlighted in bold (P<0.05).

SBP, systolic blood pressure; DBP, diastolic blood pressure; MCHC, mean corpuscular hemoglobin concentration; AST, aspartate aminotransferase; ALT, alanine aminotransferase; γ-GGT, gamma-glutamyl transpeptidase; ALP, alkaline phosphatase; HDL-cholesterol, high-density lipoprotein cholesterol; LDL-cholesterol, low-density lipoprotein cholesterol; CRP, C-reactive protein; HbA1c, hemoglobin A1c; CAP, controlled attenuation parameter; LSM, liver stiffness measurements; BSA, body surface area; IVST, interventricular septum thickness; LVPWT, left ventricular posterior wall thickness; LA, left atrial; RWT, relative wall thickness; LVEDD, left ventricular end-diastolic diameter; LVM, left ventricular mass; LVMI, left ventricular mass index; LVDD, left ventricular diastolic dysfunction.

**Table 5:** Comparison of cardiac structure according to liver fibrosis status.

Characteristics	Normal	LSM < 8.0	LSM ≥ 8.0	*p*-Value
	(N = 60)	(N = 164)	(N = 12)	
**IVST (mm)**	0.93 ± 0.11	1.04 ± 0.14	1.09 ± 0.12	**<0.001**
**LVPWT (mm)**	0.89 ± 0.1	0.98 ± 0.1	1.03 ± 0.12	**<0.001**
**LVEDD (mm)**	4.57 ± 0.4	4.73 ± 0.38	4.72 ± 0.27	**0.035**
**LA diameter (mm)**	3.21 ± 0.41	3.39 ± 0.41	3.4 ± 0.38	**0.014**
**RWT**	0.39 ± 0.04	0.41 ± 0.05	0.43 ± 0.05	**0.001**
**LVEDV (ml)**	97.3 ± 19.81	105.06 ± 20.25	103.89 ± 13.57	**0.042**
**LVM (g)**	142.02 ± 34.75	171.96 ± 37.47	181.63 ± 33.91	**<0.001**
**LVMI (BSA)**	78.06 ± 17.28	90.81 ± 18.45	92.18 ± 14.66	**<0.001**

Values are mean ( ± SD). Statistically significant values are highlighted in bold (P<0.05).

LSM, liver stiffness measurements; IVST, interventricular septum thickness; LVPWT, left ventricular posterior wall thickness; LA, left atrial; RWT, relative wall thickness; LVEDD, left ventricular end-diastolic diameter; LVM, left ventricular mass; LVMI, left ventricular mass index.

## Results

### Clinical Characteristics

In the final analysis, a total of 228 participants were stratified by presence or absence of MAFLD, and their clinical, laboratory, and metabolic characteristics are stated in **Table 2**. Of the study subjects, the mean age (40.89 ± 12.91 vs. 48.84 ± 11.64) and sex were not significantly different. The incidence of type 2 diabetes (3.5% vs. 18.7%) and hypertension (31.6% vs. 61.4%) were higher in subjects with MAFLD (all *p* < 0.05), compared to normal people. Patients with MAFLD had significantly higher BMI, waistline, white blood cell count, neutrophil count, and metabolic parameters, such as triglyceride, fasting blood glucose (FBG), serum uric acid (UA), and HbA1c and lower levels of high-density lipoprotein cholesterol (HDL-cholesterol) as compared to those without MAFLD (all *p* < 0.05).

Then MAFLD patients were divided into 3 subgroups: 108 overweight/obesity patients (overweight/obesity subgroup), 32 patients with diabetes mellitus (diabetes subgroup), and 31 lean or normal-weight patients but with at least 2 metabolic risk abnormalities (lean metabolic dysfunction subgroup), according to the above method. Overweight/obesity MAFLD patients account for the largest proportion as shown in **Figure 2A** (63.16% vs. 18.71% vs. 18.13%; *p* < 0.000, *p* for trend < 0.000). As expected, there were differences in some pronounced metabolism abnormalities among the three subgroups, such as BMI, FBG, HbA1c, uric acid, and HDL-cholesterol (all *p* < 0.05 [**Table 3**]). Measures of abdominal subcutaneous fat tissue and waist circumference were the largest in the overweight/obesity subgroup, but the diabetes subgroup had higher content in visceral fat than the other two groups (all *p* < 0.05 [**Table 3**]).

Next, an analysis of the severity of hepatic steatosis found that moderate and severe hepatic steatosis had more undesirable clinical characteristics when compared to the mild steatosis group (**Table 4**). The value of serum uric acid, for instance, increases according to the severity of MAFLD (330.00 ± 75.18 in the normal group, 351.08 ± 81.09 in mild hepatic steatosis group, 378.41 ± 84.65 in moderate hepatic steatosis group, and 391.51 ± 93.55 in severe hepatic steatosis group; *p* = 0.001).

### Association of Metabolic Dysfunction-Associated Fatty Liver Disease With Left Ventricular Diastolic Dysfunction and Cardiac Remolding (Echocardiographic Characteristics)

When compared to the normal group, more patients with MAFLD had LVDD (24.6% vs. 60.8%, *p* < 0.001 [**Table 2**]). We further assessed left diastolic function between each subgroup of MAFLD. There were no significant differences between the three groups as shown in **Table 3** and **Figure 2B** (*p* = 0.1785, *p* for trend = 0.8677). In addition, we investigated the relationship between the severity of hepatic steatosis with MAFLD and LVDD by the same method as above. Moderate-to-to severe hepatic steatosis had higher prevalence of LVDD as shown in **Table 4** and **Figure 2C** (*p* < 0.0001, *p* for trend < 0.0001).

Some markers of cardiac remolding demonstrated alterations in patients with MAFLD, manifested by increased IVST, LVPWT, LAD, RWT, LVM, and LVMI (all *p* < 0.05 [**Table 2**]). Then we analyzed the echocardiographic characteristics of MAFLD subgroups (**Table 3**). There were significant differences in echocardiographic parameters, including IVST, LVPWT, LVEDD, LA, RWT, LVEDV, LVM, and LVMI (all *p* < 0.05 [**Table 3**]). In particular, most indicators of the diabetes subgroup, compared with the other two groups, showed more obvious abnormalities. This result might indicate that different subgroups of MAFLD may affect the different degrees of the cardiac structure change. Furthermore, when the severity of hepatic steatosis was evaluated by CAP measurement, the moderate-to-to severe hepatic steatosis group showed significantly increased markers of cardiac structure, including IVST, LVPWT, LAD, and LVM when compared with the normal and mild steatosis groups (all *p* < 0.05 [**Table 4**]). What is more, we analyzed the relationship between the presence of liver fibrosis with MAFLD and cardiac structure. When MAFLD was stratified by LSM value, liver fibrosis patients had higher parameters of cardiac structure compared with non-liver fibrosis patients (all *p* < 0.05 [**Table 5**]).

### Multivariable Regression Analyses

To assess whether different subgroups and severity of MAFLD are independently related to cardiac structure, multivariable linear regression analyses were performed to adjust for clinically important factors as described above (**Tables 6**, **7**). Consequently, MAFLD patients in the diabetes and overweight/obesity subgroups were closely associated with markers of cardiac remolding in Model 1 (all *p* < 0.05 [**Table 6**]) and continuous with sight attenuation in regression coefficient after adjustment with age, sex, smoking, alcohol consumption, BMI and hypertension; the diabetes subgroups remained associated with increased IVST, LVPWT, RWT, LVM, and LVMI (all *p* < 0.05 [**Table 6**]). Further adjustment for ALT, AST, and TC in Model 3 remained significant in the diabetes subgroup [all *p* < 0.05 (**Table 6**)]. These results may provide guidance for the targeted diagnosis and treatment of high-risk patients. Similarly, to assess whether the severity of hepatic steatosis is associated with LV remolding using the above method, we found that severe hepatic steatosis patients were significantly associated with indicators of cardiac abnormality, including an increase of IVST, LVPWT, RWT, LADs, LVM, and LVMI [all *p* < 0.05 (**Table 7**)].

**Table 6:** Multivariable linear regression analysis assessing the influence of subgroups of MAFLD on echocardiographic markers of myocardial morphology.

Variable	Model 1: unadjusted			Model 2: age, sex, smoking, alcohol consumption, BMI, and hypertension adjusted			Model 3: Model 2 + ALT, AST, and TC adjusted		
	β	95% CI	*p*	β	95% CI	*p*	β	95% CI	*p*
**IVST**									
No MAFLD	1.00 (reference)			1.00 (reference)			1.00 (reference)		
Obesity subgroup	0.117	(0.074–0.16)	**0.000**	0.049	(0.001–0.097)	**0.046**	0.048	(0–0.097)	0.051
Diabetes subgroup	0.201	(0.143–0.259)	**0.000**	0.114	(0.051–0.176)	**0.000**	0.118	(0.055–0.181)	**0.000**
Leansubgroup	0.043	(−0.015 to 0.102)	0.146	0.021	(−0.036 to 0.078)	0.471	0.024	(−0.034 to 0.081)	0.415
**LVPWT**									
No MAFLD	1.00 (reference)			1.00 (reference)			1.00 (reference)		
Obesity subgroup	0.097	(0.064–0.129)	**0.000**	0.037	(0.001–0.073)	**0.046**	0.034	(−0.002 to 0.071)	0.066
Diabetes subgroup	0.153	(0.109–0.197)	**0.000**	0.085	(0.038–0.132)	**0.000**	0.083	(0.035–0.13)	**0.001**
Lean subgroup	0.001	(−0.044 to 0.045)	0.976	−0.005	(−0.049 to 0.038)	0.806	−0.005	(−0.049 to 0.038)	0.809
**LVEDD**									
No MAFLD	1.00 (reference)			1.00 (reference)			1.00 (reference)		
Obesity subgroup	0.191	(0.068–0.315)	**0.002**	−0.005	(−0.141 to 0.131)	0.942	−0.013	(−0.149 to 0.124)	0.854
Diabetes subgroup	0.193	(0.027–0.359)	**0.023**	−0.004	(−0.18 to 0.172)	0.966	0.000	(−0.178 to 0.178)	0.997
Lean subgroup	−0.014	(−0.182 to 0.154)	0.869	0.017	(−0.144 to 0.179)	0.833	0.024	(−0.138 to 0.186)	0.771
**LA**									
No MAFLD	1.00 (reference)			1.00 (reference)			1.00 (reference)		
Obesity subgroup	0.241	(0.111–0.371)	**0.000**	−0.012	(−0.151 to 0.126)	0.860	−0.023	(−0.162 to 0.117)	0.750
Diabetes subgroup	0.249	(0.073 to 0.425)	**0.006**	−0.085	(−0.265 to 0.095)	0.354	−0.105	(−0.287 to 0.077)	0.258
Lean subgroup	−0.065	(−0.243 to 0.112)	0.469	−0.122	(−0.287 to 0.044)	0.149	−0.129	(−0.295 to 0.037)	0.128
**RWT**									
No MAFLD	1.00 (reference)			1.00 (reference)			1.00 (reference)		
Obesity subgroup	0.026	(0.011–0.042)	**0.001**	0.018	(0–0.037)	**0.048**	0.018	(0–0.036)	0.055
Diabetes subgroup	0.051	(0.03–0.072)	**0.000**	0.038	(0.015–0.062)	**0.002**	0.037	(0.013–0.061)	**0.003**
Lean subgroup	0.003	(−0.018 to 0.024)	0.809	−0.004	(−0.025 to 0.018)	0.743	−0.004	(−0.026 to 0.018)	0.703
**LVEDV**									
No MAFLD	1.00 (reference)			1.00 (reference)			1.00 (reference)		
Obesity subgroup	9.572	(3.243–15.901)	**0.003**	−0.193	(−7.201 to 6.816)	0.957	−0.589	(−7.63 to 6.451)	0.869
Diabetes subgroup	9.376	(0.837 to 17.915)	**0.032**	−0.416	(−9.521 to 8.688)	0.928	−0.243	(−9.442 to 8.957)	0.959
Lean subgroup	−0.669	(−9.296 to 7.958)	0.879	0.852	(−7.489 to 9.193)	0.841	1.188	(−7.179 to 9.554)	0.780
**LVM**									
No MAFLD	1.00 (reference)			1.00 (reference)			1.00 (reference)		
Obesity subgroup	32.867	(21.667–44.067)	**0.000**	8.286	(−3.504 to 20.076)	0.167	7.427	(−4.378 to 19.232)	0.216
*Diabetes subgroup*	48.873	(33.762–63.984)	**0.000**	21.532	(6.216–36.848)	**0.006**	21.843	(6.418–37.268)	**0.006**
*Lean subgroup*	3.937	(−11.329 to 19.204)	0.612	2.632	(−11.399 to 16.664)	0.712	3.316	(−10.713 to 17.344)	0.642
**LVMI**									
No MAFLD	1.00 (reference)			1.00 (reference)			1.00 (reference)		
*Obesity subgroup*	12.197	(6.568–17.826)	**0.000**	5.426	(−0.886 to 11.737)	0.092	5.042	(−1.282 to 11.367)	0.118
*Diabetes subgroup*	22.396	(14.801–29.991)	**0.000**	12.031	(3.832–20.229)	**0.004**	12.322	(4.058–20.587)	**0.004**
*Lean subgroup*	5.261	(−2.411 to 12.934)	0.178	0.888	(−6.623 to 8.399)	0.816	1.292	(−6.224 to 8.808)	0.735

IVST, interventricular septum thickness; LVPWT, left ventricular posterior wall thickness; LA, left atrial; RWT, relative wall thickness; LVEDD, left ventricular end-diastolic diameter; LVM, left ventricular mass; LVMI, left ventricular mass index. Statistically significant values are highlighted in bold (P<0.05).

**Table 7:** Multivariable linear regression analysis assessing the influence of severity of MAFLD on echocardiographic markers of myocardial morphology.

Variable	Model 1: unadjusted			Model 2: age, sex, smoking, alcohol consumption, BMI, and hypertension adjusted			Model 3: Model 2 + ALT, AST, and TC adjusted		
	β	95% CI	*p*	β	95% CI	*p*	β	95% CI	*p*
**IVST**									
No MAFLD	1.00 (reference)			1.00 (reference)			1.00 (reference)		
MAFLD	0.119	(0.077–0.161)	**0.000**	0.047	(0.004–0.09)	**0.034**	0.047	(0.004–0.091)	**0.034**
MAFLD grade									
No MAFLD	1.00 (reference)			1.00 (reference)			1.00 (reference)		
Mild	0.094	(0.045–0.143)	**0.000**	0.037	(−0.01 to 0.085)	0.124	0.037	(−0.011 to 0.085)	0.133
Moderate	0.122	(0.055–0.188)	**0.000**	0.055	(−0.009 to 0.119)	0.093	0.056	(−0.008 to 0.121)	0.087
Severe	0.140	(0.093–0.188)	**0.000**	0.057	(0.006–0.109)	**0.029**	0.058	(0.006–0.109)	0.028
**LVPWT**									
No MAFLD	1.00 (reference)			1.00 (reference)			1.00 (reference)		
MAFLD	0.090	(0.057–0.123)	**0.000**	0.028	(−0.005 to 0.061)	0.095	0.026	(−0.007 to 0.059)	0.125
MAFLD grade									
No MAFLD	1.00 (reference)			1.00 (reference)			1.00 (reference)		
Mild	0.065	(0.027–0.103)	**0.001**	0.022	(−0.014 to 0.059)	0.228	0.018	(−0.018 to 0.055)	0.325
Moderate	0.071	(0.02–0.123)	**0.007**	0.020	(−0.028 to 0.069)	0.410	0.020	(−0.028 to 0.069)	0.412
Severe	0.116	(0.08–0.153)	**0.000**	0.040	(0.001–0.079)	**0.045**	0.039	(0–0.078)	0.051
**LVEDD**									
No MAFLD	1.00 (reference)			1.00 (reference)			1.00 (reference)		
MAFLD	0.154	(0.038–0.271)	**0.010**	0.003	(−0.117 to 0.122)	0.965	0.001	(−0.119 to 0.121)	0.985
MAFLD grade									
No MAFLD	1.00 (reference)			1.00 (reference)			1.00 (reference)		
Mild	0.148	(0.011–0.285)	**0.034**	0.044	(−0.088 to 0.176)	0.511	0.040	(−0.093 to 0.173)	0.558
Moderate	0.112	(−0.074 to 0.298)	0.238	0.007	(−0.17 to 0.183)	0.940	0.008	(−0.169 to 0.186)	0.926
Severe	0.173	(0.04–0.306)	**0.011**	−0.062	(−0.203 to 0.08)	0.392	−0.057	(−0.199 to 0.084)	0.425
**LA**									
No MAFLD	1.00 (reference)			1.00 (reference)			1.00 (reference)		
MAFLD	0.187	(0.062–0.312)	**0.004**	−0.058	(−0.181 to 0.065)	0.355	−0.068	(−0.191 to 0.056)	0.282
MAFLD grade									
No MAFLD	1.00 (reference)			1.00 (reference)			1.00 (reference)		
Mild	0.080	(−0.065 to 0.225)	0.277	−0.098	(−0.234 to 0.037)	0.152	−0.116	(−0.252 to 0.02)	0.095
Moderate	0.273	(0.076–0.469)	**0.007**	0.061	(−0.12 to 0.242)	0.508	0.053	(−0.129 to 0.234)	0.568
Severe	0.253	(0.112–0.393)	**0.000**	−0.050	(−0.195 to 0.095)	0.499	−0.052	(−0.197 to 0.094)	0.484
**RWT**									
No MAFLD	1.00 (reference)			1.00 (reference)			1.00 (reference)		
MAFLD	0.027	(0.012–0.042)	**0.000**	0.013	(−0.003 to 0.03)	0.115	0.012	(−0.004 to 0.029)	0.141
MAFLD grade									
No MAFLD	1.00 (reference)			1.00 (reference)			1.00 (reference)		
Mild	0.016	(−0.001 to 0.034)	0.064	0.007	(−0.012 to 0.025)	0.476	0.005	(−0.013 to 0.024)	0.570
Moderate	0.025	(0.001–0.048)	**0.038**	0.012	(−0.012 to 0.036)	0.339	0.012	(−0.013 to 0.036)	0.349
Severe	0.036	(0.02–0.053)	**0.000**	0.024	(0.004–0.043)	0.016	0.023	(0.004–0.042)	0.021
**LVEDV**									
No MAFLD	1.00 (reference)			1.00 (reference)			1.00 (reference)		
MAFLD	7.679	(1.703–13.654)	**0.012**	0.138	(−6.033 to 0.6.308)	0.965	0.056	(−6.145 to 0.6.258)	0.986
MAFLD grade									
No MAFLD	1.00 (reference)			1.00 (reference)			1.00 (reference)		
Mild	7.442	(0.399–14.485)	**0.038**	2.241	(−4.552 to 0.9.035)	0.516	2.018	(−4.848 to 0.8.885)	0.563
Moderate	5.979	(−3.565 to 0.15.522)	0.218	0.759	(−8.36 to 0.9.877)	0.870	0.840	(−8.308 to 0.9.987)	0.857
Severe	8.399	(1.583–15.215)	**0.016**	−3.323	(−10.62 to 0.3.974)	0.370	−3.115	(−10.434 to 0.4.203)	0.402
**LVM**									
No MAFLD	1.00 (reference)			1.00 (reference)			1.00 (reference)		
MAFLD	30.618	(19.579–41.657)	**0.000**	7.857	(−2.66 to 0.18.375)	0.142	7.528	(−3.015 to 0.18.071)	0.161
MAFLD grade									
No MAFLD	1.00 (reference)			1.00 (reference)			1.00 (reference)		
Mild	25.187	(12.288–38.086)	**0.000**	8.792	(−2.864 to 0.20.448)	0.139	7.989	(−3.754 to 0.19.731)	0.181
Moderate	25.652	(8.174–43.13)	**0.004**	7.150	(−8.495 to 0.22.796)	0.369	7.326	(−8.318 to 0.22.969)	0.357
Severe	36.801	(24.318–49.284)	**0.000**	6.763	(−5.759 to 0.19.284)	0.288	6.955	(−5.56 to 0.19.471)	0.275
**LVMI**									
No MAFLD	1.00 (reference)			1.00 (reference)			1.00 (reference)		
MAFLD	12.848	(7.434–18.262)	**0.000**	4.627	(−1.009 to 0.10.263)	0.107	4.519	(−1.135 to 0.10.173)	0.117
MAFLD grade									
No MAFLD	1.00 (reference)			1.00 (reference)			1.00 (reference)		
Mild	11.685	(5.311–18.059)	**0.000**	4.624	(−1.614 to 0.10.863)	0.145	4.279	(−2.009 to 0.10.567)	0.181
Moderate	15.493	(6.856–24.13)	**0.000**	6.893	(−1.481 to 0.15.267)	0.106	6.989	(−1.388 to 0.15.367)	0.102
Severe	13.046	(6.877–19.214)	**0.000**	3.610	(−3.092 to 0.10.312)	0.290	3.769	(−2.933 to 0.10.471)	0.269

IVST, interventricular septum thickness; LVPWT, left ventricular posterior wall thickness; LA, left atrial; RWT, relative wall thickness; LVEDD, left ventricular end-diastolic diameter; LVM, left ventricular mass; LVMI, left ventricular mass index. Statistically significant values are highlighted in bold (P<0.05)

## Discussion

The association between NAFLD and CVD has been extensively reported in the literature (20). However, the emergence of a new definition of MAFLD may produce different results in clinical studies. The main findings of our results demonstrated that patients with MAFLD exhibited significant alterations in cardiac structure and diastolic function as compared with normal people. Interestingly, different subgroups of patients with MAFLD had varying influences on cardiac function and structure. Furthermore, the prevalence of LVDD and remolding increased with the severity of hepatic steatosis. In addition, consistent with our previous studies, individuals with MAFLD had higher BMI and neutrophil counts and more markers of metabolism abnormalities (21).

In our study, the patients with MAFLD demonstrated signs of cardiac remodeling, as showed by the increased RWT and LVMI when compared with the normal group; these results are similar to previous findings on the effects of NAFLD on cardiac structure (22). Fatty liver and CVDs not only interact, but more importantly, hepatic steatosis may serve as a marker of adipose ectopic deposition in the myocardium and pericardium. Although several studies showed that subjects with NAFLD were associated with cardiac structural abnormalities and diastolic dysfunction (23, 24), these associations between the liver and heart were not sufficiently demonstrated in the different disease states of patients with hepatic steatosis. There are no studies to demonstrate the effects of different subtypes of MAFLD on cardiac structure and diastolic function. When we observed whether the adverse effects of MAFLD subtypes were different, the results suggested that the cardiac structure of MAFLD patients with different diagnostic criteria may be different, and the diabetes subgroup may have a higher risk of cardiac remodeling than lean MAFLD and overweight/obesity MAFLD subjects. Many studies have documented the high prevalence of NAFLD in type 2 diabetes patients, and there are some pathological associations between them, such as insulin resistance, chronic inflammation, and disorder of lipid metabolism (25, 26). Evidence from other studies suggested that NAFLD impaired myocardial dysfunction related to reduced myocardial glucose uptake in patients with type 2 diabetes and impaired myocardial reserve (27). A study on the association of MAFLD with diabetes, chronic kidney disease, and CVD with a 4.6-year follow-up in Chinese found that MAFLD was associated with higher risks of incident diabetes and CVD (28). T2DM can accelerate the progression of NAFLD into non-alcoholic steatohepatitis (NASH), liver fibrosis, cirrhosis, and even liver cancer. The coexistence of the two diseases can have a synergistic effect to exacerbate the damage to the heart and even increases the risk of life-threatening sequelae. In multivariate linear regression analysis, the LVM, LVEDV, and RWT were increased in the diabetes and overweight/obesity subgroups. To better evaluate whether the fatty liver is associated with cardiac remolding, age, sex, alcohol consumption, and other clinically important factors were adjusted, and the association between them remained significant. The results implied that overweight/obesity and diabetic MAFLD patients were significant risk factors for cardiac remolding. Published studies suggest that obesity adversely affects the cardiac structure and LV function before the onset of organic heart disease (18). A recent study found that NASH patients with extreme obesity were associated with LV concentric remodeling and hyperdynamic circulation (29). Like them, we have measured the waist circumstance, and subcutaneous fat, and these indicators were significantly higher in the overweight/obesity subgroup; the patterns of fat distribution may be related to the metabolic abnormalities and ectopic deposition of fat in the myocardium.

Numerous studies have shown that NAFLD is associated with LVDD (30). Many case–control trials have demonstrated the echocardiography of LVDD in NAFLD patients on whether adults, children, and adolescents are comparable to the corresponding control population without NAFLD (31–34). In our study, LVDD was significantly more prevalent in patients with MAFLD compared to normal people, in particular, in the diabetes subgroup (75.0%). There is growing evidence that hepatic steatosis and fibrosis contribute to the pathogenesis of cardiac functional abnormity (10, 35–37). Regarding the mechanisms of hepatic steatosis that adversely affect cardiac function, several hypotheses were proposed; for example, elevated epicardial fat thickness has a paracrine effect on the myocardium and contributes to altered diastolic function (38). Moreover, fatty liver disease releases proinflammatory cytokines, adhesion molecules, and procoagulant factors that promote myocardial oxidative stress, fibrosis, and deposition of advanced glycation end-products with subsequent diastolic stiffness and dysfunction (39). T2DM and fatty liver have been associated with subclinical manifestations of cardiac structure, function, and myocardial metabolism. The independent association between fatty liver and diastolic dysfunction in patients with type 2 diabetes with underlying systemic insulin resistance and hyperglycemia is noteworthy (40). Hepatic insulin resistance and even whole-body insulin are universally observed in MAFLD patients with T2DM, and it may explain the reason for more LVDD occurring in MAFLD patients with diabetes (27). Although there was no statistical significance in abnormal LV dysfunction among the three subgroups, the possibility of significant statistical significance between them cannot be ruled out with the increase in the number of subjects and the extension of follow-up time. Therefore, individualized management is required for MAFLD, which is of significant meaning for preventing complications of MAFLD (41).

Depending on the severity of fatty degeneration and fibrosis, the risk of LVDD is significantly increased (27, 35). However, the emergence of a new definition of MAFLD has hardly been investigated, as well as its dose-dependent association with severity of steatosis with higher prevalence of LVDD or cardiac remolding. In contrast, our research subjects were thoroughly examined, using the transient elastography with CAP value to quantify hepatic steatosis. Based on these results, we have found that the cardiac remodeling gradually worsened with the aggravation of hepatic steatosis and higher prevalence of LVDD in moderate to severe hepatic steatosis. What is more, most markers of cardiac remolding were significantly associated with liver fibrosis. Moreover, multivariate linear regression analysis also showed that the severity of hepatic steatosis was significantly correlated with the change in cardiac structure. Moderate-to-to severe hepatic steatosis may aggravate the insulin resistance of the liver. The liver, as the target organ and initiator organ of insulin resistance (40), secretes more proinflammatory factors that affect myocardial metabolism and cause microcirculation disorders, which leads to structural and functional disorders of the heart (42). In the moderate to severe hepatic steatosis group, obesity, hypertension, diabetes, and dyslipidemia might be important factors in exacerbating cardiac injury. In addition, a dose dependence was established between the neutrophil count and aggravation of hepatic steatosis. Therefore, reasonable treatment of MAFLD can slow down the disease progression and help reduce the occurrence of cardiovascular complications.

However, some limitations should also be noted. First, we have used hepatic elastography to assess the severity of hepatic steatosis rather than liver biopsies. Liver biopsies are the gold standard for the diagnosis of fat accumulation in the liver and allow the assessment of the evidence of inflammation and ballooning degeneration of hepatocytes, which are the most important histological features for predicting disease progression of MAFLD, but this approach is invasive and not suitable for most patients. Second, we did not have evaluated objective measures of the participants’ physical activity. A study pointed out the positive effect of physical activity on fibrosis and CVD in patients with NAFLD (43). This is one of the potential factors that can be used to conduct future prospective studies to clarify the effect of physical activity on cardiac function and structure in patients with MAFLD. Third, LSM results suggested that a small number of our study participants have fibrosis, and we will further elaborate on it in future studies. Therefore, these limitations should be taken into account when interpreting our study findings and analyzing the results objectively.

In conclusion, our results revealed the significant association of MAFLD with LVDD and cardiac remolding in the general population, which indicated the clinical significance of MAFLD as a potential risk factor for cardiovascular events. What is more, we further demonstrated that diabetic, overweight, and moderate to severe steatosis patients were most closely associated with abnormalities of cardiac structure and function, which is of great significance for the precise intervention of MAFLD. We also suggest that medical professionals need to screen patients with MAFLD for cardiac injury to prevent disease progression.

## Data Availability Statement

The original contributions presented in the study are included in the article/supplementary material. Further inquiries can be directed to the corresponding author.

## Ethics Statement

The studies involving human participants were reviewed and approved by Hangzhou Normal University Affiliated Hospital. The patients/participants provided their written informed consent to participate in this study.

## Author Contributions

ZY was responsible for the study concept and design. DP did the statistical analysis and wrote the manuscript. MW, JS, LS, YZ, WZ, CC, JT, CW, JN, WW, and JJ collected clinical specimens. All authors critically revised the paper and approved the final version.

## Funding

This study was funded by the Health Science and Technology Project of Zhejiang Province, China (2020KY216) and the Hangzhou City Health Science and Technique Program (OO20190126).

## Conflict of Interest

The authors declare that the research was conducted in the absence of any commercial or financial relationships that could be construed as a potential conflict of interest.

## Publisher’s Note

All claims expressed in this article are solely those of the authors and do not necessarily represent those of their affiliated organizations, or those of the publisher, the editors and the reviewers. Any product that may be evaluated in this article, or claim that may be made by its manufacturer, is not guaranteed or endorsed by the publisher.
